# Wand of the Alchemist

**Published:** 1898-07

**Authors:** 


### WAND OF THE ALCHEMIST.—WHAT THE MAN OF SCIENCE MAY SECURE FROM COAL TAR.

[From the popular English Magazine, *The Humanitarian.*]

The wand of the alchemist becomes more wondrous in its transforming powers every day. No tale in the Arabian Nights, no story of the wondrous treasures taken by mystic power from magic nutshells surpasses what science is doing to-day.

Science, the wizard of the century, touches with his fairy wand the black, viscid coal tar from the gas retorts and coal becomes not ony a source of light and heat, but an arsenal of colors, a buffet of dainty tastes, a medicine chest for suffering humanity, a store-house of new foods and exquisite perfumes, a source of powerful explosive for war and so many other miraculous powers that the telling challenges credence. From the 140 pounds of gas tar in a ton of coal, science to-day makes aniline dyes, numbering over 2,000 distinct shades, many of them being of exquisite delicacy, so that vegetable dyes are almost displaced.

Of medicines, antiseptic, hypnotic and fever-allaying preparations, it furnishes antipyrine, *ammonol*, antifebrin, asparol, carbolic acid, diuretin, dulcin, euphorin, exalgine, hypnol, malarin, napthalin, phenatecin, phenol, salol, sulfonal, trional hylene and a host of others.

It furnishes perfumes—queen of the meadows, cinnamon, bitter almonds, camphor, wintergreen and thymol. It has given to the world bellite and picrite, two powerful explosives. It supplies flavoring extracts that duplicate the taste of currants, raspberries, pepper, vanilla, etc. It is the housekeeper's ally, with benzine and naphtha, the insecticides. It supplies the farmer with ammonial fertilizers. It has given to the photographer his two developers, hydroquinone and eikonogen. It makes the anatomist its debtor for a most wonderful stain for tissues. It contains the substance which tints the photographer's lens. It yields paraffin, creosote, pitch; material for artificial paving; saccharin, a substance 300 times sweeter than sugar, and saccharin-amide, still sweeter; lampblack, material for red ink, lubricating oils, varnish, resin, almost our entire supply of ammonia, and hundreds of other things—all these science brings forth from this coal tar.

By means of its products—this waste that surpassed its uselessness only by its offensiveness—we can make preserves without either fruit or sugar, perfumes without flowers, and coloring matter without animal or vegetable aid of any description.

